# Origin of metallic behavior in NiCo_2_O_4_ ferrimagnet

**DOI:** 10.1038/srep15201

**Published:** 2015-10-15

**Authors:** Yugandhar Bitla, Yi-Ying Chin, Jheng-Cyuan Lin, Chien Nguyen Van, Ruirui Liu, Yuanmin Zhu, Heng-Jui Liu, Qian Zhan, Hong-Ji Lin, Chien-Te Chen, Ying-Hao Chu, Qing He

**Affiliations:** 1 Department of Materials Science and Engineering, National Chiao Tung University, Hsinchu, 300, Taiwan; 2 National Synchrotron Radiation Research Center, Hsinchu 300, Taiwan; 3 Institute of Physics, Academia Sinica, Taipei 105, Taiwan; 4 Department of Material Physics and Chemistry, University of Science and Technology Beijing, Beijing 100083, China; 5 Department of Physics, Durham University, Durham DH1 3LE, United Kingdom

## Abstract

Predicting and understanding the cation distribution in spinels has been one of the most interesting problems in materials science. The present work investigates the effect of cation redistribution on the structural, electrical, optical and magnetic properties of mixed-valent inverse spinel NiCo_2_O_4_(NCO) thin films. It is observed that the films grown at low temperatures (*T* *<* 400 °C) exhibit metallic behavior while that grown at higher temperatures (*T* > 400 °C) are insulators with lower ferrimagnetic-paramagnetic phase transition temperature. So far, n-type Fe3O4 has been used as a conducting layer for the spinel thin films based devices and the search for a p-type counterpart still remains elusive. The inherent coexistence and coupling of ferrimagnetic order and the metallic nature in p-type NCO makes it a promising candidate for spintronic devices. Detailed X-ray Absorption and X–ray Magnetic Circular Dichroism studies revealed a strong correlation between the mixed-valent cation distribution and the resulting ferrimagnetic-metallic/insulating behavior. Our study clearly demonstrates that it is the concentration of Ni^3+^ions and the Ni^3+^–O^2−^Ni^2+^ double exchange interaction that is crucial in dictating the metallic behavior in NCO ferrimagnet. The metal-insulator and the associated magnetic order-disorder transitions can be tuned by the degree of cation site disorder via growth conditions.

Complex oxides with spinel structure comprise a family of materials that exhibit a wide range of electronic, magnetic, optical and catalytic properties through the variation of cations on tetrahedrally and octahedrally coordinated sites. NiCo_2_O_4_ (NCO) exhibits an inverse spinel structure (space group: 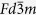) and has a ferrimagnetic Curie temperature of 673 K^1^. NCO displays better electronic conductivity (higher than conventional binary transition metal oxides), rich redox reactions, higher electrochemical activity, low-cost, high availability, environmental-friendliness and hence, it has been widely used in various technological applications such as photo-detector^2^, infrared-transparent electrode^3^,^4^; electrocatalysis of oxygen^5^,^6^,^7^,^8^,^9^, methanol^10^, CO^11^, CH_3_OH-H_2_O_2_^12^, urea^13^, and most importantly, NCO with different morphologies has also been extensively exploited in supercapacitor applications^14^. However, the key issues pertaining to NCO are (i) charge and/or spin state distribution of Co and Ni are far from clear^5^,^15^,^16^,^17^,^18^,^19^,^20^,^21^,^22^,^23^ and (ii) low thermal stability (disintegrates into oxides of Ni and Co when prepared at temperatures higher than 400 °C^1^,^5^). Cation distribution in spinels can influence many fundamental properties such as saturation magnetization, exchange couplings, magnetic ordering temperatures and electronic conductivity which result primarily from the valence state preferences of the transition metal ion for different interstitial sites^24^. In fact, predicting and understanding the cation distribution in spinels has been one of the most interesting and persistent problems in materials science. The cation distribution in mixed-valent inverse spinel NCO can be expressed in terms of a general formula as 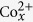 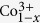 [Co^3+^ 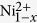 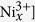 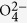 (0 *<* x *<* 1) wherein A-site (tetrahedral, T_*d*_) is occupied by high spin (HS) Co^2+^ (^4^A_2_, e^4^ 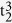, S = 3/2) and Co^3+^ (^5^E, e^3^ 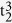, S = 2) while Ni^2+^ (^3^A_2*g*_, 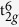 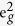, S = 1), low spin (LS) Ni^3+^ (^2^E_*g*_, 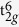 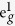, S = 1/2) and diamagnetic LS Co^3+^ (^1^A_1*g*_, 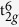 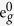, S = 0) occupy the B-site (octahedral, O_*h*_). Out of all cations, Ni^3+^ ions exhibit a strong Jahn-Teller distortion. Large dispersion in the value of x reported in bulk NCO^5^,^15^,^16^,^17^,^18^,^19^,^20^,^21^,^22^,^23^ in the above mentioned formula is due to the fact that different synthesis methods and conditions yield spinel NiCo_2_O_4_ with similar nominal composition but with significantly different cation distributions^22^. Irrespective of x, the above formula predicts a saturation moment of 2 *μ*_*B*_.

Up to date, most of the investigations have been on polycrystalline materials and the reports on single crystalline NCO are very few^4^,^25^,^26^,^27^,^28^,^29^. Epitaxial thin films of spinel NiCo_2_O_4_ films grown at lower temperatures (<450 °C) are reported to be ferrimagnetic with metallic characteristics while those grown at higher temperatures are non-magnetic and insulating^26^. The intrinsic coexistence and coupling of ferrimagnetic order and the metallic nature in NCO along with its infrared transparency makes it a unique candidate for spintronic applications. The NCO serves as p-type analogue to Fe_3_O_4_ that has been widely used as a conducting layer for the spinel thin films based structures. Understanding the origin of such intriguing behavior is crucial to advancing their practical applications in spintronic devices. In the present study, by using pulsed laser deposition (PLD) technique, high quality single crystalline NCO films with larger growth window displaying a variety of structural, magnetic and electronic phases are obtained. A comparative study on single crystalline spinel NCO films representative of metallic and insulating phases is undertaken to understand the correlation between cation (T_*d*_/O_*h*_)-site disorder and observed intriguing behaviors. We exploit the extreme sensitivity of x-ray absorption spectroscopy and x-ray magnetic circular dichroism techniques to the local electronic structure of NCO to quantify the cation distribution with varying oxidation states and also their relative magnetic moment orientations. Thus, allowing us to elucidate the underlying mechanism responsible for observed disparities in different phases. We show that both the systems have mixed-valent inverse spinel structure but with different cation distribution or disorder which directly correlates with their optical, structural, magnetic and transport properties. Our study paves an elegant route to tune the fascinating phenomena by modulating the interplay among various interactions and triggers significant interest to explore new multifunctional systems.

## Results

### Crystal Structure

**Figure 1:**
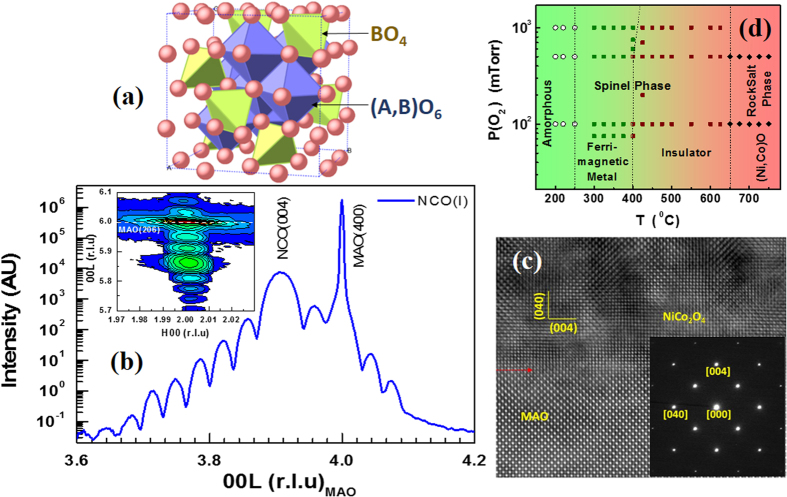
(**a**) Schematic of AB_2_O_4_ inverse spinel with Oxygen anions connecting the BO_4_ tetrahedra and A/BO_6_ octahedra. (**b**) Room temperature x-ray diffractogram of insulating NiCo_2_O_4_ film grown on MgAl_2_O_4_(100) substrate. Corresponding Reciprocal Space Map around the (206) reflection is shown in the inset. (**c**) Cross-sectional HRTEM image of NCO/MAO with selected area diffraction patterns along the [100] zone axis and (**d**) The P-T phase diagram of NCO.

**Figure 2:**
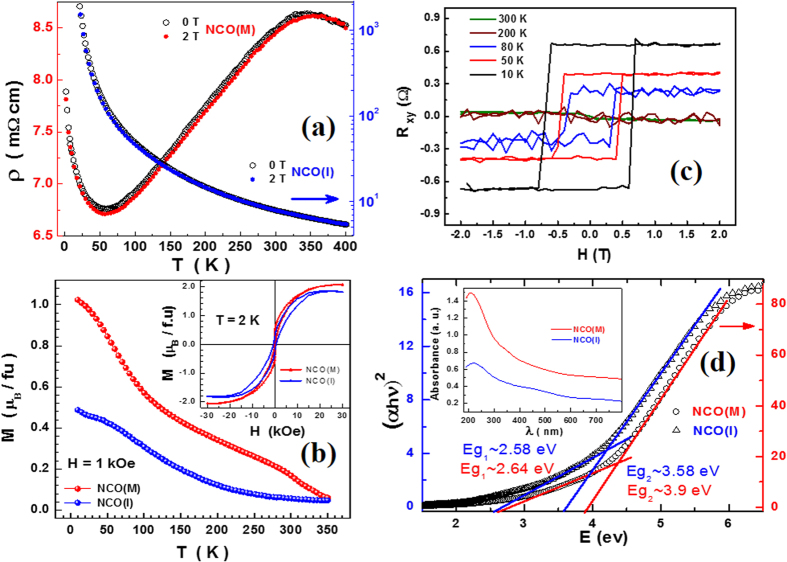
(**a**) Resistivity as a function of temperature in zero-field and in-field (2 T) for NCO(M) and NCO(I). (**b**) Thermal variation of magnetization at a static field of 1 kOe. The inset shows the magnetic hysteresis loop measured at 2 K. (**c**) Anomalous hall resistance as a function of magnetic field at various temperatures and (**d**) The (*αhν*)^2^ − *hν* plots from Optical absorption spectra (inset) of metallic and insulating NCO.

The AB_2_O_4_ spinel crystal structure is composed of the face-centered cubic lattice of O^2−^ ions with the cations A and B occupying the tetrahedral and octahedral sites in the lattice. In a normal spinel, the A cations occupy one eighth of the tetrahedral sites and the B cations occupy half of the octahedral sites; while in an inverse spinel, all the A cations occupy octahedral sites with the B cations equally distributed between the octahedral and tetrahedral sites. Figure 1(a) shows a schematic of AB_2_O_4_ inverse spinel crystal structure with oxygen anions connecting the BO_4_ tetrahedra and A/BO_6_ octahedra. With more than four hundred films deposited under various growth conditions, the grown films are found to follow the phase diagram (frequency of actual experimental points is reduced for the sake of clarity) with idealized sharp phase boundaries shown in Fig. 1(d). As the growth temperature is increased, different structural, magnetic and electronic phases such as amorphous phase (*T* *<* 250 °C), spinel ferrimagnet with decreasing *T*_*C*_ that display metallic (250–400 °C) and insulating (400–650 °C) behaviors and highly insulating Rock salt phase (*T* > 650 °C) are encountered. Temperature decides not only the rate of cation-disorder/inversion but also their redistribution with mixed valencies. Moreover, substrate-induced strain will also serve as an additional degree of freedom to further tune the above phase diagram. Two representative spinel phases that display the contrasting metallic (M) and insulating (I) behaviors, henceforth, referred to as NCO(M) and NCO(I), respectively, are undertaken for the present study. The epitaxial growth of NCO(I) film on MAO(001) substrate is evident from the room temperature high-resolution x-ray diffraction (HR-XRD) measurements shown in Fig. 1(b) (Fig. S1 in supporting information for NCO(M)). The strong peaks of (004) reflections in the *θ*–2*θ* scan confirm the high crystalline quality of the films with c-orientation without any secondary phases. The clear interference pattern, around the NCO(004) reflection indicates the presence of high crystal quality and sharp interface between the film and substrate. It is worth to note that NCO(M) and NCO(I), have different out-of-plane lattice constants that can be identified from XRD peak positions. The estimated out-of-plane lattice constants of NCO(M) and NCO(I) are 8.216 *Å* and 8.273 *Å*, respectively, higher than the bulk value (8.116 *Å*). The film thickness of ~50 nm and ~30 nm for NCO(M) and NCO(I), respectively were estimated from the distinctive oscillations around the (004) reflection. For a given set of growth parameters, the strain state of the NCO films was reported to be independent of thin film thickness as high as 700 nm^25^. The reciprocal space map around the (206) diffraction peak taken on NCO(I) sample is shown in the inset of Fig. 1(b) (NCO(M) shown in Fig. S1 in supporting information). The clear single NCO(206) peak directly below MAO(206)peak indicates high crystalline quality while the same horizontal positions of NCO(206) and MAO(206) peaks imply that the films are fully compressively strained and have identical in-plane lattice constants as the substrate (a = 8.083 *Å*). However, the expansion in the out-of-plane lattice with a fixed in-plane lattice constant results in increased unit cell volumes of 536.38 *Å*^3^ (+0.33%) for NCO(M) and 540.25 *Å*^3^ (+1.1%) for NCO(I) compared to the bulk value (534.60 *Å*^3^). This gets reflected in the functional properties that are sensitive to structure, inter-atomic bond lengths and bond angles as evident from the distinct optical, electrical and magnetic behaviors of NCO(M) and NCO(I) shown in Fig. 2. The reason for such intriguing behaviors will be elaborated later. Assuming the Poisson ratio of *ν* = 0.30, as is the case of most of the isotropic materials^30^, the calculated bulk lattice constants for NCO(I) and NCO(M) are 8.185 *Å* and 8.155 *Å* which result in a higher in-plane compressive strain of 1.25% in NCO(I) than 0.88% in NCO(M). Moreover, the increase in out-of-plane lattice constant and hence, the unit cell volume of NCO departing from bulk values with growth temperature exhibited a contrasting trend compared to other spinel oxide thin films^31^. Possible origins for such a variation can be due to the formation of oxygen or cation vacancies or increased strain with growth temperature. We believe that this has to do with the cation redistribution with temperature as evident from our XAS analysis. Further microstructure characterization of the NCO thin film was carried out by transmission electron microscopy (TEM). Figure 1(c) shows a cross-sectional high resolution TEM image of NCO(I) sample (NCO(M) is shown in Fig. S2 in supporting information) imaged along the [100] zone axis of MAO. It suggests that the film is epitaxial with a high crystallinity and exhibits a well-defined interface with the MAO substrate. No misfit dislocations or other defects were observed at the interface. The corresponding selected area electron diffraction pattern from the film and partial substrate display sharp and pure spots, as shown in the inset, confirms the coherent growth of the NCO phase on the spinel substrate. Also, no spare spots often representing the intermediate phase/domains were observed. A cube-on-cube orientation relationship can be obtained as: NCO(040)
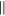
MAO(040) and NCO[100]
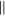
MAO[100] consistent with HR-XRD data.

### Magneto-transport, magnetic and optical properties

Resistivity as a function of temperature in zero- and 2 T-magnetic field for NCO(M) and NCO(I) samples display contrasting behaviors in Fig. 2(a). As the temperature is reduced, NCO(M) exhibits an insulator-metal transition at ~320 K and re-enters into an insulating state below 50 K clearly displaying a resistivity minimum similar to perovskite manganites. Such a minimum in resistivity was argued to arise due to disorder-induced quantum interference effect^25^. NCO(I) is insulating down to 50 K with an increase in resistivity by three orders of magnitude. The magnetic field had a very little effect on the resistivity resulting in a negative magnetoresistance of ~1% for NCO(M) and ~8% for NCO(I) at 2 T. The thermal variation of in-plane magnetization of NCO(M) is roughly twice that of NCO(I) at a static field of 1 kOe as depicted in Fig. 2(b). The magnetic transition temperature, *T*_*C*_, calculated from the dip in the temperature derivative of magnetization resulted in two peaks at 60 K and 305 K for NCO(M) while a single transition at 90 K for NCO(I). The magnetic transition temperatures of NCO(M) directly correlate with those seen in resistivity suggesting an underlying intrinsic common mechanism governing the metal-insulator and magnetic order-disorder transitions. The estimated Curie constant (C) and the Curie temperature (*θ*) based on Curie-Weiss fit, *χ*(*T*) = *C*/(*T* − *θ*), for metallic sample are 3.27(17) × 10^−3^ *μ*_*B*_ K/Oe and 295(2) K while that of insulating samples are 1.08(2) × 10^−2^ *μ*_*B*_ K/Oe and 110(2) K. The positive sign of *θ* suggests the presence of net ferromagnetic interactions below the ordering temperature. *θ*/*T*_*C*_ ~1 implies that only nearest neighbor interactions are important from two-sublattice theory^32^. The calculated paramagnetic moments are 3.46 *μ*_*B*_ and 4.69 *μ*_*B*_, respectively, while those expected from Co^2+^ [Co^3+^ Ni^3+^]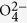 (x = 1.0) and Co^3+^ [Co^3+^ Ni^2+^]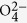 (x = 0.0) are 3.74 *μ*_*B*_ and 5.1 *μ*_*B*_, respectively. The inset of Fig. 2(b) shows the magnetic hysteresis measured at 2 K with saturation magnetization values of 2 *μ*_*B*_ for NCO(M) and 1.8 *μ*_*B*_ for NCO(I) closer to expected value of 2 *μ*_*B*_ but higher than the reported values^15^,^16^,^23^,^26^. There is buckling of magnetic hysteresis loop at origin for NCO(M) indicating the presence of strong antiferromagnetic correlations at low temperatures which can result in localized charge carriers. This observation supports the low-temperature up turn in resistivity. Furthermore, NCO exhibits anomalous Hall effect (AHE) and this is the first time such a phenomenon is reported in NCO. Hall resistivity as a function of magnetic field, R_*xy*_(H), for NCO(I), displayed in Fig. 2(c), exhibits a large coercivity of 8 kOe at 10 K that decreases with temperature and vanishes above the magnetic transition temperature. Such an observation is made on NCO(M) also and its obvious to assign inherent ferrimagnetic order responsible for the observed AHE. However, a more careful study is needed to further understand its implications and will be dealt elsewhere. Additionally, NCO samples in these two phases also exhibit different optical properties. NCO(M) shows higher optical absorption than NCO(I) as shown in inset of Fig. 2(d). The estimated optical energy gaps from (*αhν*)^2^ − *hν* plots for metallic film (2.64 eV and 3.9 eV) are slightly higher than the insulating one (2.58 eV and 3.58 eV), suggesting similar long-range ordering for both cases. The Density Functional theory calculations^28^ on NCO indicated larger band gap values for the ferrimagnetic structure as compared to the non-magnetic counterpart. Therefore, it is reasonable to believe that a local disorder cation distribution is the key to modify the electronic structure and thus the band gap of the material^28^.

### Cation distribution by XAS

**Figure 3:**
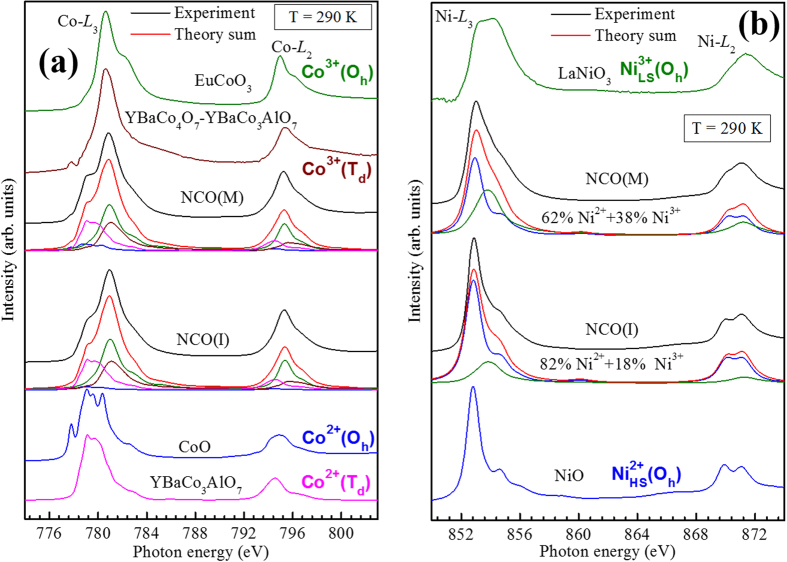
Comparison of (**a**) Co L_2,3_ and (**b**) Ni L_2,3_ of NCO(M) and NCO(I) with site simulation result. The spectra of references are also presented.

Having established the distinct behaviors in NCO(M) and NCO(I), we now investigate their local electronic structures so as to understand the origin for such behaviors. XAS and XMCD are powerful tools to unveil the local electronic structures of transition-metal oxides as XAS is extremely sensitive to the symmetry of the initial state, i.e., the spin, orbital, and valence states of the ions while XMCD provides valuable information on specific alignment of spin states. Spectral features vary with oxidation states, coordination environment and the relative abundance. To obtain detailed information about the valence states, orbital occupation, and spin states of Ni and Co ions in NCO, we have performed the Co-*L*_2,3_ and Ni-*L*_2,3_ XAS experiments. The Co and Ni XAS spectra of NCO(M) and NCO(I) are shown in Fig. 3(a,b). The reference spectra of CoO^33^ as O_*h*_ HS Co^2+^, EuCoO_3_^34^ as O_*h*_ LS Co^3+^, YBaCo_3_AlO_7_ as T_*d*_ HS Co^2+^, YBaCo_4_O_7_-YBaCo_3_AlO_7_^35^ as T_*d*_ HS Co^3+^, NiO as a O_*h*_ HS Ni^2+^ and LaNiO_3_ as O_*h*_ LS Ni^3+^ are used to simulate the site and valence states of Ni and Co using the configuration-interaction cluster calculations^36^,^37^. The higher energy shift of the average peak weight in NCO(M) than in NCO(I), as clearly evident at Ni-L_2_ edge, suggests the increase of the average oxidation state of the Ni ions. The distribution of charge and spin states of Co and Ni ions as determined from the XAS analysis are 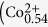 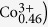 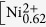

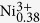
 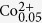

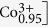
 for the metallic NCO(M) and 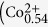 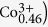 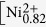

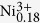
 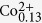

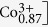
 for insulating NCO(I) where in parenthesis represent the tetrahedral sites occupation and the brackets that of the octahedral sites. As the valence and spin states of Co ions in both samples are similar it is implicit that Ni ion distribution is decisive in distinct transport behaviors. Recently, a detailed polarized Raman study suggested mixed-valent cation distribution on octahedral and tetrahedral sites for ferrimagnetic and metallic NCO thin films while an ideal inverse spinel distribution, Co^3+^ [Ni^2+^ Co^3+^]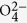, for non-magnetic and insulating thin films^27^. Our XAS results not only provide a direct and solid evidence to the scenario of mixed-valent cation distribution of NCO but also establishes a clear-cut distinction between the metallic and insulating phases in terms of their relative concentrations. Thus, enabling us to understand the mechanism responsible for such contrasting behaviors. To the best of our knowledge, no such information on NCO is available till date. The inherent coexistence and coupling among the magnetic order and electronic conductivity forms a charming playground, provides a means to manipulate the intriguing properties and thus tailor desired functionalities in the material system.

### Spin and orbital moment of Cations by XMCD

**Figure 4:**
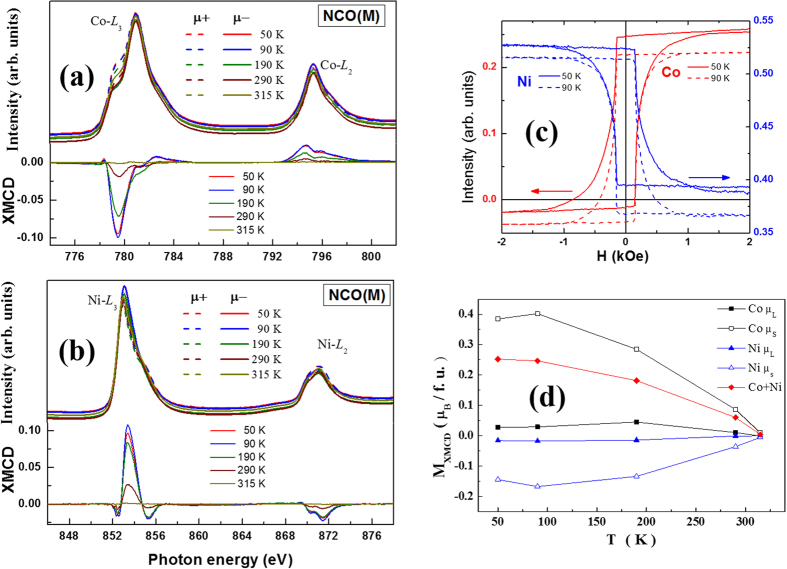
XAS and (bottom) XMCD spectra at the (**a**) Co and (**b**) Ni edges of NCO(M) at various temperatures. (**c**) Element resolved XMCD hysteresis loop at the cobalt edge(red) and nickel edge (blue) for NCO(M) at 50 K and 90 K. (**d**) Estimated spin and orbital moment contributions of Co and Ni along with the net total moment at various temperatures.

In order to get further insights about individual spin and orbital moment contributions of Ni and Co ions occupying different sites in NCO(M), we have performed XMCD experiments at various temperatures. Pairs of x-ray absorption spectra (*μ*_+_/*μ*_−_) are measured with circularly polarized x-rays with ±0.1 T magnetic fields applying to the samples. The XMCD spectra arise from the difference between the *μ*_+_ and *μ*_−_ spectra. The main objective of the present work is to evolve a mechanism that leads to metallic behavior in NCO ferrimagnet and hence, all the measurements are focused on NCO(M). According to the XMCD spectra, the magnetic moment of Ni in NCO is anti-parallel to that of Co at all the temperatures, as shown in Fig. 4(a,b). Thus, we believe that the conduction at the octahedral sites is not between the Co and Ni ions but among the Ni ions only. Moreover, most of the Co ions at octahedral site is in diamagnetic trivalent state that do not contribute to either magnetic or transport behavior while the magnetic moment contribution of Co ions is dominated by those present in tetrahedral sites. The XMCD signal at 315 K is almost zero as it is above *T*_*C*_ and thus, the magnetic moment is close to zero for both Co and Ni. The *M*−*H* loops at 50 K and 90 K shown in Fig. 4(c) also highlight the anti-parallel moment contributions of Ni and Co ions. The same magnetic coercivity of Co and Ni was observed and indicates strong coupling between them. In order to compare our element-specific XMCD data with the total magnetization in Fig. 2(b), we analyzed the results by applying the XMCD sum rules^38^,^39^,^40^. The temperature variation of net magnetic moment along with individual spin and orbital magnetic moments of Co and Ni is shown in Fig. 4(d). The number of holes in all the initial states is determined by the cluster calculations presented in Fig. 3. It is interesting to note that XMCD signals of Co and Ni at 50 K are both smaller than those at 90 K; however, the rate of decrease of moment of Ni is much faster than that of Co ions so that the net magnetic moment increases with decreasing temperature as seen in Fig. 4(d). This is related to the charge localization at Ni ions, due to possible charge/orbital ordering, as reflected below resistivity minimum at 50 K in Fig. 2(a).

## Discussion

The electronic conductivity in NCO was argued^41^ due to the formation of σ^*^(*e*_*g*_) band via intervening oxygen ion through strong covalent interaction between low spin Co^3+^–Ni^3+^ in the octahedral site assuming the distribution Co^2+^[Ni^3+^Co^3+^]O_4_. The detailed XAS analysis suggests the presence of more Ni^3+^ ions at the octahedral sites in NCO(M) than in NCO(I). This also explains the increased volume of NCO(I) than NCO(M) as more of Ni^2+^ ions of larger ionic radii occupy the octahedral sites in the former. The basic interactions mediating through intervening O-ions that results in the observed magnetic behavior as well as provide pathways for conduction are –J_*AB*_: Co^2+^–Ni^2+^, Co^2+^–Ni^3+^, Co^2+^- Co^3+^, Co^3+^–Ni^2+^, Co^3+^-Ni^3+^, Co^3+^–Co^3+^, J_*BB*_: Ni^2+^–Ni^2+^, Ni^2+^–Ni^3+^, Ni^2+^–Co^3+^, Ni^3+^–Ni^3+^, Co^3+^–Co^3+^, Ni^3+^–Co^3+^, and J_*AA*_: Co^2+^–Co^2+^, Co^2+^–Co^3+^, Co^3+^–Co^3+^. It is the interplay between these interactions that decides the observed intriguing properties of NCO. This interplay can be fine-tuned via temperature and oxygen partial pressure during the growth of NCO leading to the cation redistribution among T_*d*_/O_*h*_ sites. Particularly, temperature decides not only the rate of cation migration or degree of inversion^24^ but also their oxidation states. Among the interactions mentioned above, the antiferromagnetic superexchange interactions among homovalent ions (Ni^2+^–Ni^2+^, Ni^3+^–Ni^3+^, Co^2+^–Co^2+^ and Co^3+^–Co^3+^) tends to localize the charge carriers while the ferromagnetic double-exchange interactions (Co^2+^-Ni^3+^, Co^3+^-Ni^2+^ and Ni^2+^–Ni^3+^) delocalizes charge carriers and thus, their competition dictates the conduction mechanism. Among the latter, intersite Co^2+^–Ni^3+^ ↔ Co^3+^–Ni^2+^ and Co^3+^–Ni^2+^ ↔ Co^2+^–Ni^3+^ interactions are less effective than intrasite (O_*h*_-site) Ni^2+^–Ni^3+^ ↔ Ni^3+^-Ni^2+^ (degenerate) interactions resulting in a *σ*(e_*g*_) band via intervening oxygen ion in deciding the metallic behavior in NCO due to the antiparallel spin orientations of Co and Ni ions. In the spinel structure, the B-site octahedra share their edges so that both direct B-B interaction as well as 90° B-O-B interaction dominate the electronic behavior. The charge carriers are delocalized within these B-O-B chains that form pyrocholre lattice in the unit cell. As the trivalent Ni ion concentration (x) increases, the range of delocalized carriers as well as carrier density increases and beyond a threshold concentration (x_*c*_) these conducting chains percolate throughout the lattice resulting in the metallic behavior. Hence, we believe that this percolative picture involving the increased range of ferromagnetic-metallic double exchange interactions with decreasing growth temperature provides a correct description of the system. However, it should be noted that Ni^3+^ is an active Jahn-Teller (JT) ion and hence, the mechanism of the metal-insulator transition may involve a strong electron-lattice interaction via JT polarons.

In conclusion, we have effectively employed the XAS and XMCD techniques to discern the local electronic structures of NCO in the metallic and insulating phases. XAS/XMCD analysis revealed a strong correlation between cation distribution and the material properties. Particularly, it is the concentration of Ni^3+^ (x) ions that is crucial in dictating the metallic behavior in NCO ferrimagnet. The value of x can be tuned via growth conditions as evident in the present study. For x > x_*c*_ (0.3) the system is metallic and insulating otherwise. Lowering the growth temperature will ensure that x is always above x_*c*_ thereby result in metallic nature while higher temperature localizes more number of charge carriers due to large number of divalent Ni ions and drives the system towards insulating state. Hence, the metal-insulator transition in NCO is merely due to competing double exchange and super exchange interactions where in growth temperature plays a decisive role in tilting the intricate balance in favor of one of them.

## Methods

Epitaxial NiCo_2_O_4_ thin films were grown on MgAl_2_O_4_(100) substrate by PLD at temperatures spanning 200 °C to 700 °C and in the oxygen partial pressures of 10–1000 mTorr. High-resolution symmetry and asymmetry X-ray diffraction techniques were used to verify the epitaxial relationship between the film and substrate in beamline BL13B and BL17B at the National Synchrotron Radiation Research Center (NSRRC), Taiwan. The interface micro-structure was further studied by cross-sectional transmission electron microscopy. Magnetic measurements were carried out using a Superconducting Quantum Interference Device (Quantum Design SQUID). Field-cooled (FC) magnetization was taken from 2 K–350 K with an applied magnetic field of 0.1 T (1000 Oe). Hysteresis measurements were carried out at 2 K in magnetic fields between ±3 T. A Quantum Design physical property measurement system (PPMS) was used for temperature dependent resistivity measurements using a four-probe or Van der Pauw geometry. The soft X-ray absorption spectroscopy experiments were carried out at the BL11A Dragon beamline of the NSRRC in Taiwan. The Co-*L*_2,3_ and Ni-*L*_2,3_ spectra were recorded in the total electron yield (TEY) mode with a photon energy resolution of 0.015 eV. CoO and NiO single crystals were measured simultaneously in a separate chamber to calibrate the photon energy.

## Additional Information

**How to cite this article**: Bitla, Y. *et al.* Origin of metallic behavior in NiCo_2_O_4_ ferrimagnet. *Sci. Rep.* **5**, 15201; doi: 10.1038/srep15201 (2015).

## Supplementary Material

Supplementary Information
