# Supplementary material for: Origin of metallic behavior in NiCo2O4 ferrimagnet
Source: Sci Rep. 2015 Oct 15;5:15201. doi: 10.1038/srep15201 (PMC4606736; doi:10.1038/srep15201)
Supplement: Supplementary Information [file srep15201-s1.pdf]

Supplementary Information

Origin of Ferri-metallic behavior in $NiCo_2O_4$

Yugandhar Bitla[†], Yi-Ying Chin[‡], Jheng-Cyuan Lin[§], Chien Nguyen Van[†], Ruirui Liu[||], Yuanmin Zhu[||], Heng-Jui Liu[†], Qian Zhan[||], Hong-Ji Lin[‡], Chien-Te Chen[‡], Ying-Hao Chu[†,§], and Qing He[#]

[†]Department of Materials Science and Engineering, National Chiao Tung University, Hsinchu, 300, Taiwan

[‡]National Synchrotron Radiation Research Center, Hsinchu 300, Taiwan

[§]Institute of Physics, Academia Sinica, Taipei 105, Taiwan

[||]Department of Material Physics and Chemistry, University of Science and Technology Beijing, Beijing 100083, China

[#] Department of Physics, Durham University, Durham DH1 3LE, United Kingdom

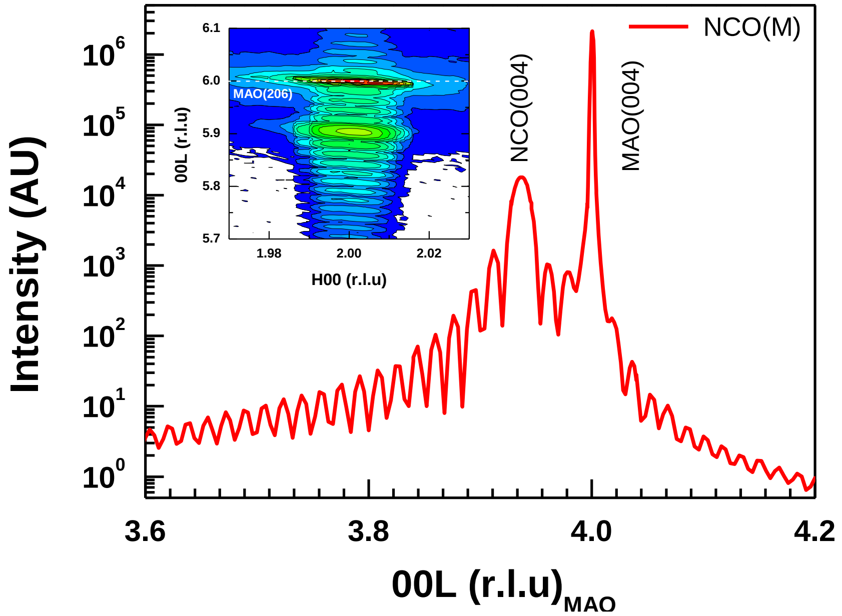

Figure S1. Room temperature x-ray diffractogram of metallic $NiCo_2O_4$ film grown on $MgAl_2O_4(100)$ substrate. Corresponding Reciprocal Space Map around the (206) reflection is shown in the inset.

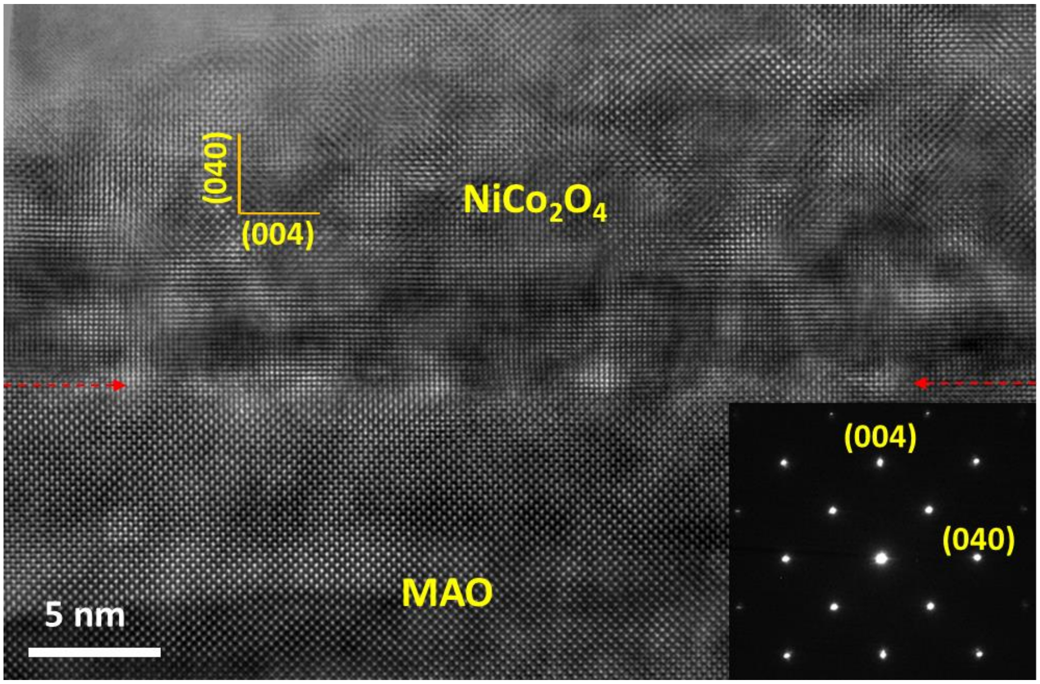

Figure *S*2. Cross-sectional HRTEM image for the $NiCo_2O_4$ (NCO(M)) films grown on $MgAl_2O_4$ substrate. The corresponding selected area diffraction patterns of the NCO films and MAO substrate along the [100] zone axis.
